# Triple bag hermetic technology for controlling a bruchid (*Spermophagus* sp.) (Coleoptera, Chrysomelidae) in stored *Hibiscus sabdariffa* grain

**DOI:** 10.1016/j.jspr.2016.05.004

**Published:** 2016-10

**Authors:** L. Amadou, I.B. Baoua, D. Baributsa, S.B. Williams, L.L. Murdock

**Affiliations:** a Institut National de la Recherche Agronomique du Niger (INRAN), BP 240, Maradi, Niger; b University of Maradi, Faculty of Agronomy and Environmental Sciences, BP 465, Maradi, Niger; c Department of Entomology, Purdue University, West Lafayette, IN, 47907, USA

**Keywords:** *Hibiscus sabdariffa*, Hermetic, Triple bag, Postharvest, Storage, *Spermophagus* sp.

## Abstract

We assessed the performance of hermetic triple layer Purdue Improved Crop Storage (PICS) bags for protecting *Hibiscus sabdariffa* grain against storage insects. The major storage pest in the grain was a bruchid, *Spermophagus* sp.. When we stored infested H. *sabdariffa* grain for six months in the woven polypropylene bags typically used by farmers, the *Spermophagus* population increased 33-fold over that initially present. The mean number of emergence holes per 100 seeds increased from 3.3 holes to 35.4 holes during this time period, while grain held for the same length of time in PICS bags experienced no increase in the numbers of holes. Grain weight loss in the woven control bags was 8.6% while no weight loss was observed in the PICS bags. Seed germination rates of grain held in woven bags for six months dropped significantly while germination of grain held in PICS bags did not change from the initial value. PICS bags can be used to safely store *Hibiscus* grain after harvest to protect against a major insect pest.

## Highlights

• *Hibiscus sabdariffa* is produced by low resource farmers in Sub-Saharan Africa.
• Stored *Hibiscus* grain is attacked by a bruchid of the genus *Spermophagus*.
• *Spermophagus* infestations severely damage the grain and reduce germination.
• Storage in triple layer hermetic PICS bags prevents losses due to *Spermophagus*.

## 1 Introduction

*Hibiscus sabdariffa* L. (Malvaceae) is grown in tropical and subtropical regions of the world (http://scialert.net/fulltext/?doi=ajcs.2012.103.112&org=11, Babatunde, 2003; http://scialert.net/fulltext/?doi=ajcs.2012.103.112&org=11, Tindal, 1986). It is cultivated for its stems, calyces, leaves, and grain (Babatunde and Mofoke, 2006). In West Africa *Hibiscus* leaves, calyces, and grains are used as vegetables to impart aroma to sauces and to prepare a popular drink. The dried red calyces are the basis for a tea or juice known as ‘bissap’ (Senegal) or ‘da bilenni’ (Côte d’Ivoire, Mali, Burkina Faso) (FAO, 2004). Seeds of *H. sabdariffa*, are known for their rich nutrient content and are used to produce an oil, while the plant itself is known for its medicinal properties (Mahadevan et al., 2009). The grain is nutritious, containing an average of 26% protein, 20% fat, and 40% total sugars (Cissé et al., 2009). Demand for *Hibiscus* has steadily increased over the years. Currently approximately 15,000 metric tons enter international trade annually (http://www.fao.org/fileadmin/user_upload/inpho/docs/Post_Harvest_Compendium_-_Hibiscus.pdf).

In the Sahel of Africa women process the seeds by cooking and fermenting to produce “soumbala”, a fortifier used in many sauces. *H. sabdariffa* grain production occurs during the rainy season, which extends from June to October. Grain stores kept by producers constitute the main supply through the year, with storage lasting from 3 to 10 months. During storage there is risk of pest insect attack if the grain is not protected. In recent years research and development efforts have concentrated on increasing food production in Sub-Saharan Africa (Anankware et al., 2012). Despite its economic and nutritional importance for families, *Hibiscus* grain storage has received little attention and farmers, aware of potential losses to insects, often use unregistered and potentially dangerous pesticides to protect their grain. We found a bruchid present in *Hibiscus* seeds stored by African farmers that we identified as belonging to the genus *Spermophagus*. Unlike other bruchids which use legume seeds as larval host food, bruchine beetles of the genus *Spermophagus* utilize seeds of morning glories (Convulvacea) and mallows (Malvaceae: Malvoidea) (Kergoat et al., 2015). *Hibiscus sabdariffa* is a domesticated mallow.

Purdue Improved Crop Storage (PICS) bags consist of two layers of high density polyethylene (HDPE) enclosed by a polypropylene bag (see below); they provide excellent protection of cowpea grain against bruchid seed beetles in West Africa (Murdock et al., 2012, Baoua et al., 2012, Baoua et al., 2013). They are likewise effective in protecting other stored grains against insect pests, including (1) maize attacked by the larger grain borer (Njoroge et al., 2014); (2) Bambara ground nut against bruchids (Baoua et al., 2014b), and; (3) mung beans and pigeonpea (Baoua et al., 2014b, Mutungi et al., 2014) attacked by bruchids. PICS bags, on the other hand, were found not to be effective in controlling cassava chips (Hell et al., 2014) infested with larger gain borer. We sought to determine if PICS bag could protect *H. sabdariffa* grain against its main storage pest while maintaining seed viability.

## 2 Materials and methods

Our experiments used naturally infested *Hibiscus sabdariffa* grain and were carried out at the INRAN entomology laboratory in Maradi, Niger. All work was done at uncontrolled ambient room temperature, which ranged from 28 to 39 °C, and at ambient relative humidity (5–30% r.h.). Relative humidity was measured using EL USB 2 data logger devices (Lascar, Erie, PA, USA). Grain moisture content was determined by weighing samples before and after oven drying and ranged from 8 to 10%. We used triple layer PICS bags manufactured in 2011 by Lela Agro (Kano, Nigeria). The PICS bag consists of two separate high-density polyethylene (HDPE) bags with 80 μm wall thickness, one fitted inside the other, and both of which were enclosed in a woven polypropylene bag to enable handling. Details for using PICS bags are found on the PICS website (http://www.ag.purdue.edu/ipia/pics/Pages/Home.aspx).

Naturally infested grain of *H. sabdariffa* produced in two different years (2010 and 2011) was purchased at Maradi market and thoroughly mixed to create a uniform infestation throughout the lot.

The first treatment, in four replicates, employed 50 kg PICS bags. These were filled with 40 kg of infested *Hibiscus* grain from the pooled source. The control treatment consisted of two replicate single woven polypropylene bags filled with the same grain.

We collected the following data:

(1) Initial and final infestation levels: These were determined at the beginning of the experiment by collecting 3 samples of 500 g grains per bag, each selected randomly. Each sample was sieved using 1.5–2.0 mesh screen sieves to separate the living adults. Pupae were counted as adults. The same measurements were made on the grain after storage. When bags were opened for sampling at 2 and 4 months, they were immediately reclosed after the sample was taken.
(2) Initial and final damage level: One hundred seeds were randomly selected from each sample, weighed, and the number of seeds with eggs and holes determined by counting.
(3) Germination: Tests were conducted in the field on a research plot. For each treatment, 4 rows of 25 seeds were sown. The plot was irrigated daily and the number of germinated plants recorded at 7 days after sowing.
(4) Storage and evaluation: Bags were opened after 2, 4 and 6 months and the above analyses were done to evaluate the degree of bruchid infestation, grain damage, and any effects of storage on germination.
(5) Gas concentrations: O_2_ and CO_2_ levels in each bag were determined using a Mocon PAC Check^®^ Model 325 Headspace analyzer (Mocon, Minneapolis, MN). Measurements were made daily at 12:00 PM local time over the first 20 days.
(6) Bag conditions: At the end of the experiments, the two HDPE liners were examined for holes and abrasions as well as for other physical changes.

Statistical analysis: Means of O_2_ and CO_2_ concentrations were compared using the *t*-test. Insect density and damage levels were compared using Analysis of Variance (ANOVA) followed by Least Significant Difference (LSD) tests. Statistical analysis was done with SPSS software (Statistical Package for the Social Sciences), produced by IBM SPSS, Inc. (Chicago, Illinois).

The experiments were conducted during the six month period from January 14 to July 14, 2012.

## 3 Results

**Fig. 1:**
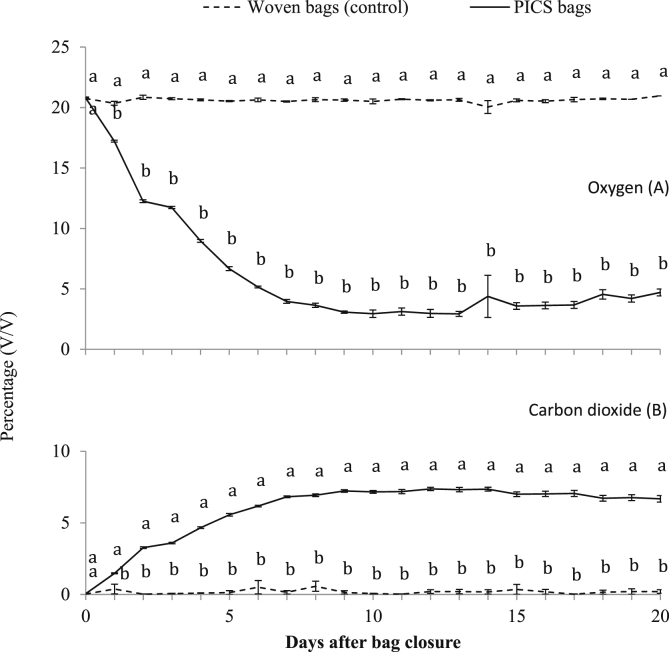
Daily levels of O_2_ and CO_2_ in PICS bags and woven bag containing naturally infested *H. sabdariffa* seeds.

Oxygen levels differed significantly between treatments as soon as one day after the bags were closed, with means of 17.2 ± 0.1% by volume (v/v) for PICS bags after 24 h compared to 20.4 ± 0.2% (v/v) for the controls (t = 20.21; df = 4; P < 0.001). By the 20th day (Fig. 1) O_2_ had fallen to 4.7 ± 0.3% (v/v) in the PICS bags while remaining unchanged compared to ambient at 21.0 ± 0.0% (v/v) for the control (t = 37.86; df = 4; P < 0.001). CO_2_ levels differed between treatments by the second day after closing the bags with an average of 1.5 ± 0.0% (v/v) in the PICS bags and 0.4 ± 0.3% (v/v) in the control treatments (t = −5.14; df = 4; P < 0.01). By the 20th day CO_2_ levels were 6.7 ± 0.2% (v/v) in PICS bags and 0.2 ± 0.1% (v/v) in the controls (t = −18.45; df = 4; P < 0.001).

*Spermophagus* sp. was the only insect species we observed in the *H. sabdariffa* grain. Its identity was confirmed based on morphology and photographs sent to the Natural History Museum of Paris in France.

The number of living *Spermophagus* sp. adults found in the PICS bags after 2, 4, and 6 months of storage was the same as at the outset of the experiment, whereas in the woven bags, the number of bruchids was 11 times higher after 2 months, 13 times higher after 4 months, and 33 times higher after 6 months (Table 1).

The frequency of seeds carrying *Spermophagus* sp. eggs, and those exhibiting emergence holes was not different in the PICS bags from that initially noted at the start of the experiment. By contrast, after 6 months of storage grain held in the control bags had 9–11 times as many eggs and emergence holes as they did initially. After two and four months of storage, the weight of 100 seeds in the PICS bags and control bags did not differ significantly from the initial value (Table 2). However, by six months of storage in PICS bags, the 100 seed weight was similar to what was initially noted while in the control bag we observed a weight loss of 8.6%.

The *Hibiscus* grain we used had an initial germination rate of 42.1 ± 1.3%. After six months of storage, it was not significantly different in PICS bags (44.1 ± 1.3%) from what was noted initially. However, grain stored in the woven control bags exhibited a germination rate of only 28.8 ± 2.7%, a decrease of 53% (F = 18.47, df = 2/69, P < 0.001).

Inspection of the HDPE liners of the PICS bags used in these experiments revealed few or no holes and few or no “abrasions”, i.e., small frosty spots on the surfaces of the bags. Such holes and abrasions are not uncommon when cowpeas infested by the cowpea bruchid are stored in PICS bags.

## 4 Discussion

The *Spermophagus* sp. observed here is a known but little studied bruchid pest of stored *Hibiscus* seeds. While bruchids are most commonly associated with legumes (Fabaceae), they are also known to attack non-leguminous species of the families of Convolvulaceae and Malvaceae (Kergoat et al., 2015). *Spermophagus* negligens has been reported in *H. sabdariffa* seed stocks seized by USA APHIS airport authorities in Philadelphia in 1997 and in Baltimore in 2012 (http://www.cbp.gov/newsroom/local-media-release/2013-02-26-050000/baltimore-cbp-intercepts-first-port-seed-beetle).

The O_2_ level in PICS bags dropped over the first three weeks while CO_2_ rose. By contrast, there was little change in the levels of these two gases in the woven control bags. This is simply explained by the fact that the triple layer PICS bags are practically hermetic and greatly retard gas movement across the bag walls. Woven bags, on the other hand, have a single porous wall, and as insects living in the grain held in these bags grow, develop and reproduce, the O_2_ they consume is replaced by diffusion from the environment and the CO_2_ they produce likewise diffuses out, resulting in little change in the concentrations of the two gases within the woven bags.

The drop in O_2_ and rise in CO_2_ observed in PICS bags leads to *Spermophagus* sp. mortality and failure of its population to grow. Our results point towards cessation of egg production and development, as well as larval and adult mortality. Similar changes in gas composition and arrest of population development have been observed with stored maize (De Groote et al., 2013) and Bambara ground nuts (Baoua et al., 2014a) as well as with other stored crops and pests. The simplest hypothesis to explain these results is that prolonged restriction in available O_2_ not only suppresses the insect population growth but leads to the reduction in the supply of water because of the inadequate supply of O_2_, leading to eventual insect death by desiccation (Murdock et al., 2012).

Our results establish that hermetic conditions arrest *Spermophagus* sp. population growth. The numbers of adults and emergence holes in *Hibiscus* grain held in PICS bags for six months were far less than observed in the woven control bag. After six months of storage the *Spermophagus* sp. population was 91.2 adults per 500 g of grain held in woven bags, while in PICS bags the pest density was less than 2 living insects per 500 g. This finding is consistent with that obtained by Mutungi et al. (2014) who tested PICS bag for storing mung beans and pigeon peas.

After six months of storage in woven bags the mean *H. sabdariffa* grain weight losses was 8.6%. It is interesting that this is substantially lower than the weight loss observed by Baoua et al. (2014a) when bruchid-infested Bambara ground nut was stored in woven bags.

Regarding the near absence of abrasions and holes in the HDPE liners, it appears that *Spermophagus* sp. is less damaging to HDPE compared to Callosobruchus maculatus on cowpea (Baoua et al., 2012) and larger grain borer (P. truncatus Horn) on maize (Baoua et al., 2014b). PICS bags become even more economical if they can be reused (Jones et al., 2011). Bag reuse depends on the degree of damage seen as holes, abrasions, broken seals and tears. When no holes, abrasions or more serious damage is seen, the bag will continue to protect grain for additional uses. PICS bags used for cowpea in Niger are used, on average, for three storage seasons (Baributsa et al., 2014).

*H. sabdariffa* grain germination was generally low at the beginning of the experiment, that is, less than 50%. This is explained by the fact that our experiments used infested grain produced in both 2010 and 2011 and so was older on average and had been exposed to *Spermophagus* sp. prior to beginning the experiments. We observed an average of 44.1% germination of this grain after six month of storage in PICS bags, a rate comparable to the initial rate of 42.1% and not statistically significant. Exposure of the grain to lowered levels of oxygen in PICS bags evidently had no effect on seed viability, just as has been observed in the case of cowpea (Baoua et al., 2013); mung bean (Mutungi et al., 2014); Bambara ground nut (Baoua et al., 2014a); and maize (Baoua et al., 2014b).

## Figures and Tables

**Table 1:** Infestation of *H. sabdariffa* grain by *Spermophagus* sp. adults in triple bags and woven bags filled with naturally infested grain and stored for six months.

Periods	Treatments	Number of samples analyzed	Live adults of *Spermophagus* sp. per 500 g of *H. sabdariffa* seeds
After 2 months	Initial infestation	18	2.8 ± 5.7 a
	PICS bag	12	1.8 ± 3.1 a
	Woven bag	6	30.5 ± 4.0 b
	Anova		(F = 94.31, *df* = 2/33, *p* < 0.001)
After 4 months	Initial infestation	18	2.8 ± 5.7a
	PICS bag	12	0.7 ± 1.1a
	Woven bag	6	37.0 ± 8.8 b
	Anova		(F = 106.81, *df* = 2/33, *p* < 0,001)
After 6 months	Initial infestation	18	2.8 ± 1.7 a
	PICS bag	12	0.1 ± 0.1 a
	Woven bag	6	91.2 ± 52.3b
	Anova		(F = 6.35, *df* = 2/33, *p* < 0.001)

Means followed by the same letter are not significantly different.

**Table 2:** Infestation and damage to *H. sabdariffa* grain by *Spermophagus* sp. stored in 50 kg triple bags and control woven bags. Bags were filled with naturally infested grain and stored for six months.

	Treatments	Number of samples analyzed	Observation per 100 grains of *H. sabdariffa*		
			Grains bearing eggs of *Spermophagus* sp.	Seed with emergence holes of *Spermophagus* sp.	Weight (g)
After 2 months	Initial infestation	36	4.8 ± 3.7a	3.3 ± 2.2a	3.5 ± 0.5a
	PICS bag	24	4.9 ± 2.9a	3.5 ± 2.4a	3.5 ± 0.1a
	Woven bag	12	27.0 ± 10.8b	11.8 ± 2.9b	3.5 ± 0.1a
	Anova		(F = 76.80, *df* = 2/69, p < 0.001)	(F = 34.69, *df* = 2/69, p < 0.001)	(F = 0.13, *df* = 2/69, p = 0.87)
After 4 months	Initial infestation	36	4.8 ± 3.5a	3.3 ± 2.2a	3.5 ± 0.5a
	PICS bag	24	6.0 ± 5.3a	4.3 ± 2.9a	3.5 ± 0.2a
	Woven bag	12	21.4 ± 10.6b	7.7 ± 5.7b	3.4 ± 0.1a
	Anova		(F = 34.96, *df* = 2/69, p < 0.001)	(F = 7.17, *df* = 2/69, p < 0.05)	(F = 0.30, *df* = 2/69, p = 0.74)
After 6 months	Initial infestation	36	4.79 ± 0.60a	3.3 ± 0.4a	3.5 ± 0.1a
	PICS bag	24	4.83 ± 0.49a	3.9 ± 0.4a	3.5 ± 0.0a
	Woven bag	12	44.61 ± 8.78b	35.4 ± 6.7b	3.2 ± 0.1b
	Anova		(F = 39.14, *df* = 2/69, *p* < 0.001)	(F = 43.77, *df* = 2/69, *p* < 0.001)	(F = 7.08, *df* = 2/69, *p* < 0.001)

Means followed by the same letter are not significantly different (LSD, 5%).
